# Data on evaluation of proficiency for the US-CAB curriculum

**DOI:** 10.1016/j.dib.2018.02.021

**Published:** 2018-02-16

**Authors:** Wan-Ching Lien, Shu-Hsien Hsu, Kah-Meng Chong, Shyh-Shyong Sim, Meng-Che Wu, Wei-Tien Chang, Cheng-Chung Fang, Matthew Huei-Ming Ma, Shyr-Chyr Chen, Wen-Jone Chen

**Affiliations:** a Department of Emergency Medicine, College of Medicine, National Taiwan University and National Taiwan University Hospital, Taipei, Taiwan; b Department of Emergency Medicine, Far-Eastern Memorial Hospital, New Taipei City, Taiwan

## Abstract

Data presented in this article relates to the research article entitled “US-CAB protocol for ultrasonographic evaluation during cardiopulmonary resuscitation: validation and potential impact” (Lien et al., in press). The article provides data regarding proficiency of the 10 emergency residents attending the US-CAB curriculum. Assessments included immediate evaluation at the end of training and re-evaluation 6 months later. A written test, and the ultrasound image acquisition were required in the immediate evaluation The re-evaluation included the written test and performance on the same healthy volunteer.

**Specifications Table**

**Table:** 

Subject area	*Human science*
More specific subject area	*Ultrasound training*
Type of data	*Tables*
How data was acquired	*Prospective observational*
Data format	*Analyzed*
Experimental factors	*Time to achieve ultrasound images*
Experimental features	*The 10 emergency physicians attended the US-CAB curriculum. Assessments included immediate evaluation at the end of training and re-evaluation 6 months later. A written test, and the ultrasound image acquisition were required in the immediate evaluation The re-evaluation included the written test and performance on the same healthy volunteer.*
Data source location	*National Taiwan University Hospital, Taipei, Taiwan*
Data accessibility	*The data are with this article.*
Related research article	*US-CAB protocol for ultrasonographic evaluation during cardiopulmonary resuscitation: validation and potential impact. Resuscitation (in press).*

**Value of the Data**

1. The data can provide the information for designing ultrasound training course.
2. The data serve as a benchmark for human performance in focused ultrasound training.
3. The data allow assessment of training proficiency immediately and after a 6-month interval.
4. The data enable investigating the effects of memory retention on ultrasound performance.
5. Analysis of the data provide the information regarding human performance in emergency situations.

## 1 Data

A novel US-CAB protocol was proposed for systematic evaluation of the circulation(C)-airway (A)-breathing (B) status during resuscitation [1], [2]. The emergency medicine residents attended the US-CAB training curriculum in August 2015. The data are based on the assessments of these participants with ultrasound performance immediately and with a 6-month interval after the training.

## 2 Experimental design, materials, and methods

### 2.1 Study population

The 10 junior emergency physicians (EPs), the 1st and 2nd year of residents of the Department of Emergency Medicine of the National Taiwan University Hospital, who had attended the basic emergency ultrasound training, attended the US-CAB training curriculum in August 2015 (Table 1). The curriculum is a half-day course, including one-hour didactics, and three-hour small-group rotatory hand-on training on live healthy model volunteers. The ratio of the instructor to participant is less than 1:5. The instructors are the expert sonographers, board-certified in emergency ultrasound (US) [2].

**Table 1:** The characteristics of the participants.

Variables	Participants
	(*N* = 10)
Age (years)	28.9 ±2.1
Male gender, *n* (%)	4 (40%)
Previous C-A-B ultrasound experience, *n* (%)	
Cardiac ultrasound	
No experience	0
<30 cases	10 (100%)
Tracheal ultrasound	
No experience	10 (100%)
<30 cases	0
Lung ultrasound	
No experience	0
<30 cases	10 (100%)

### 2.2 Course assessments

Course assessments included immediate evaluation and re-evaluation. A written test, and the US image acquisition were required in the immediate evaluation at the end of the training. The written test included 10 multi-choice questions, comprising of ALS knowledge and interpretation of still C-A-B images before and after completion of the course. The participants were asked for to produce adequate views of each C-A-B category, recorded by the DVD recorder, on the same healthy volunteer. The videos were edited that the faces of the participants were covered, and reviewed blindly by one board-certified expert in emergency US. The time to achieve the views was measured from positioning of the probe on the skin to maintaining an adequate image.

The re-evaluation was conducted with an interval of 6 months after the training, including the same written test and US performance on the same healthy volunteer.

A US scanner (SSA-550A, Toshiba, Tokyo, Japan) equipped with 2–5 MHz curvilinear transducers, was used.

### 2.3 Statistics

Data analysis was performed using the SAS software (SAS 9.4, Cary, North Carolina, USA). Paired t-test and Student's t-test were employed for the continuous data, as well as Chi-square test for the categorical data. A p-value of less than 0.05 was considered statistically significant.

The demographic data of the participants were shown in the Table 1. The pre- and post-course written test results, and the time to obtain adequate views were listed in the Table 2.

**Table 2:** The evaluation of the US-CAB curriculum.

	Immediate	After 6 months	During CPR [1]
	evaluation		
Pre-test score	6.9 ±1.1		
Post-test score	9.3 ±0.9†	9.7 ±0.5	
Cardiac (seconds)	19.7 ±12.7	12.2 ±2.4‡	9.0 ±1.4§
Airway (seconds)	10.8 ±4.6	8.3 ±1.7‡	7.5 ±1.5§
Breathing (seconds)			
Left	13.0 ±8.1	11.6 ±2.1	8.5 ±2.0§
Right	12.6 ±7.5	12.6 ±2.6	7.5 ±1.8§

All variables were expressed as mean ±SD.

† *P* < 0.0001 when comparing the post-test score with the pre-test score

‡ *P* < 0.001 when comparing the 6-month performance with the immediate evaluation.

§ *P* < 0.001 when comparing the performance during CPR with the 6-month performance
